# Trends in exercise for hypertension: a bibliometric analysis

**DOI:** 10.3389/fcvm.2023.1260569

**Published:** 2023-10-23

**Authors:** Yan Lou, Ning Sun, Min Zhang, Yongzhen Qiu, Jie Wang, Jiajia Chen

**Affiliations:** ^1^ Rehabilitation Medicine Center and Institute of Rehabilitation Medicine, West China Hospital, Sichuan University, Chengdu, China; ^2^ Key Laboratory of Rehabilitation Medicine in Sichuan Province, Sichuan University, Chengdu, China; ^3^ Department of Nursing, Zhejiang Zhoushan Tourism and Health College, Zhoushan, China; ^4^ Department of Nursing, Lishui Central Hospital and Fifth Affiliated Hospital of Wenzhou Medical College, Lishui, China; ^5^ Department of Nursing, Hangzhou Normal University, Hangzhou, China; ^6^ Department of Nursing, the First Affiliated Hospital of Zhejiang Chinese Medical University (Zhejiang Provincial Hospital of Traditional Chinese Medicine), Hangzhou, China

**Keywords:** non-pharmacological intervention, hypertension management, research hotspots, CiteSpace, high blood pressure

## Abstract

**Objective:**

To investigate development trends and research hotspots of exercise for hypertension research and provide researchers with fresh perspectives for further studies.

**Materials and methods:**

Articles and reviews regarding exercise and hypertension spanning May 1st 2003 to May 18th 2023 were retrieved from the Web of Science Core Collection (WOSCC) database. VOSviewer and Citespace were mainly used to perform and visualize co-authorship, co-citation, and co-occurrence analysis of countries, institutions, authors, references and keywords in this field.

**Results:**

A total of 1,643 peer-reviewed papers were identified, displaying a consistent increasing trend over time. The most prolific country and institution were Brazil and University of Sao Paulo, respectively. And we identified the most productive author was lrigoyen, Maria Claudia C, while Pescatello Linda S was the most co-cited author. *Journal of hypertension* was the most prominent journal, and *Hypertension* was the journal which was the most co-cited. And this field can be divided into 3 research themes: exercise interventions for hypertension, age-specific relevance of exercise for hypertension, and the global burden of hypertension and the role of exercise. According to the result of keywords analysis, epidemiological information, types of exercise, target population, mechanism, and study design are significant research areas. “Resistance training”, “adults”, and “heart rate variability” were identified as the major future research foci.

**Conclusions:**

The findings offer a scientific insight into exercise for hypertension research, presenting researchers with valuable information to understand the current research status, hotspots, and emerging trends for future investigation.

## Introduction

Hypertension, commonly known as high blood pressure, is characterized as a persistent elevation in blood pressure with an unclear etiology, thereby amplifying the risk for cerebral, cardiac, and renal events (1). The precise medical definition of hypertension varies among clinicians due to differential guideline recommendations. Some suggest that hypertension is defined by consistent readings exceeding 130/80 mmHg, whereas others advocate for a threshold of 140/90 mmHg (2). In the year 2010, hypertension was reported in approximately 31.1% of the global adult population. Projections predicted a rise of 60% by 2025, resulting in a prevalence of 29.2%, or around 1.56 billion individuals (3, 4). By 2010, hypertension had emerged as the predominant single risk factor contributing to the global disease burden, accountable for 9.4 million deaths and 7.0% of global disability-adjusted life years, with impacts across all socio-economic strata (5, 6). The economic implications of hypertension are also considerable. In 2003, the cumulative direct and indirect expenses associated with the management of hypertension in the US were estimated to be USD 50.3 billion (7).

The challenge with hypertension lies in its often-asymptomatic nature, making it difficult for individuals to realize their condition. If left uncontrolled over extended periods, hypertension can precipitate severe medical conditions, such as heart failure, myocardial infarction, stroke, vision complications, and kidney disease (1). Therefore, patient monitoring is a crucial aspect of hypertension management. It's typically controlled by medication and lifestyle changes, with exercise playing a crucial role. Numerous therapeutic exercise regimens have been implemented as interventions in hypertension management (8). In recent years, a multitude of studies have demonstrated that aerobic and resistance exercise, coupled with flexibility and balance exercises, can augment the quality of life for patients with hypertension (9, 10). These improvements manifest in various ways such as enhancing cardiovascular system function, optimizing blood pressure control, and reducing the risk of apoplexy (11, 12).

Bibliometric analysis, a robust quantitative research methodology, offers an objective framework to trace the intellectual evolution and structural composition of a specific research domain (13). It has been widely utilized to probe developmental trends and hotspots within various areas of publication (14–16). To our understanding, despite the steady increase in publications relating to exercise in the hypertension research field, no bibliometric analysis has yet been conducted. Findings derived from such bibliometric studies can enable investigators to pinpoint current research concerns, thereby guiding future research directions (17). Owing to its considerable advantages, the application of bibliometric methods holds significant value in the domain of exercise for hypertension research.

In this study, our objective is to conduct a thorough bibliometric analysis of research concerning exercise for hypertension. The intention is to offer a comprehensive assessment of the evolution within this field. By meticulously examining past achievements, evaluating the present state, and projecting potential future directions, we aspire to illuminate the landscape of exercise for hypertension research.

## Materials and methods

### Data acquisition

**Figure 1:**
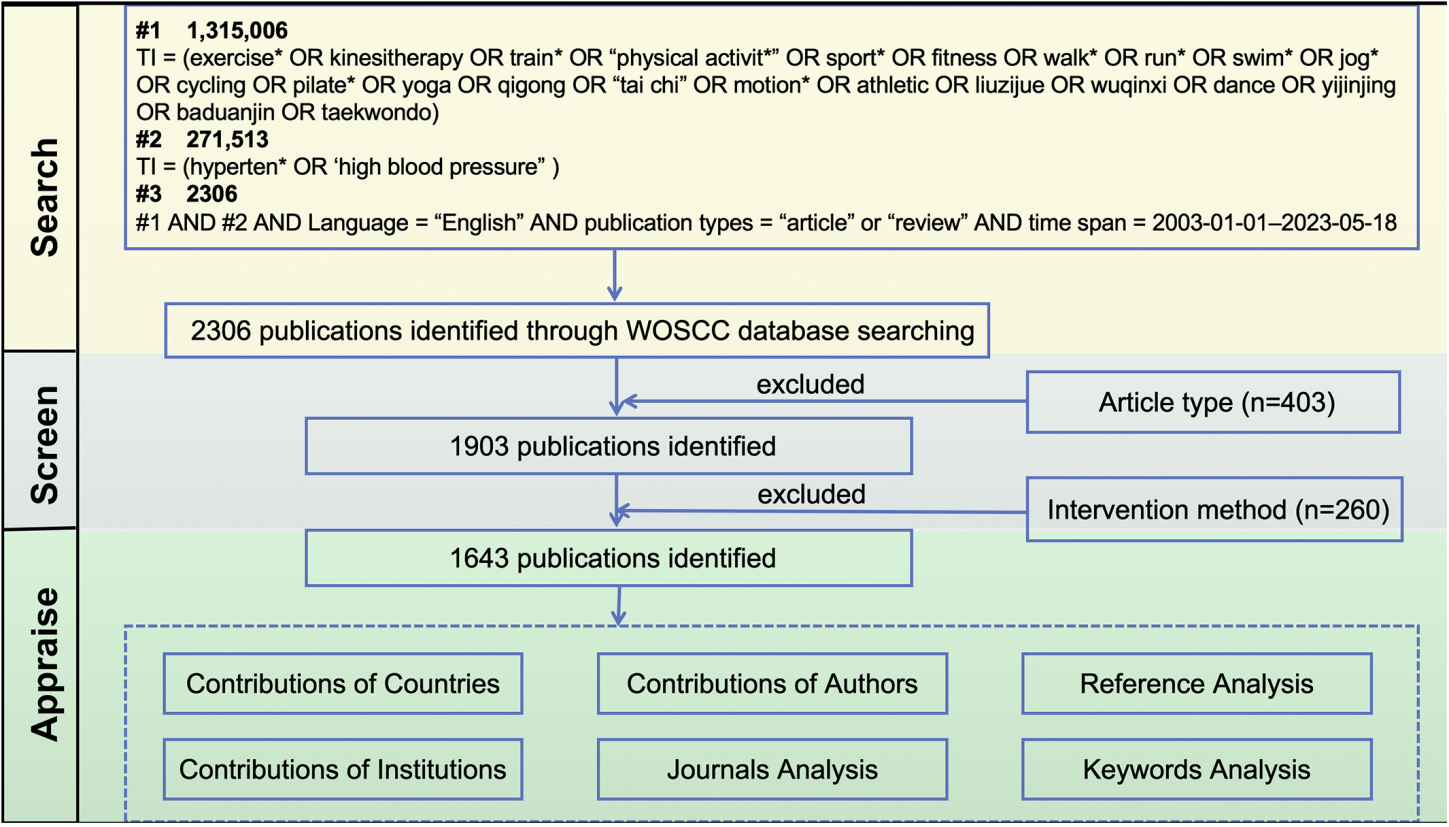
Flowchart of the publications selection.

The Web of Science Core Collection (WOSCC) database was searched extensively for publications relating to exercise for hypertension on 19 May 2023, which is frequently used and accepted for scientific or bibliometric studies. A peer-reviewed and supervised search term development process was conducted under the guidance of an expert with ten years of information retrieval experience. The data search strategy was “TI = (exercise* OR kinesitherapy OR train* OR “physical activit*” OR sport* OR fitness OR walk* OR run* OR swim* OR jog* OR cycling OR pilate* OR yoga OR qigong OR “tai chi” OR motion* OR athletic OR liuzijue OR wuqinxi OR dance OR yijinjing OR baduanjin OR taekwondo) AND TI = (hyperten* OR “high blood pressure”) AND Language = “English”. In this study, the publication types were restricted to “article” or “review”, time span = 2003.01.01–2023.05.18 (Figure 1).

### Data analysis and visualization

The data, which incorporates fully recorded and cited references, was downloaded from WOSCC. In addition to the Online Analysis Platform of Literature Metrology (https://bibliometric.com/), VOSviewer 1.6.17, and CiteSpace 6.1.R3, this information was also imported into several analysis tools. The key metrics adopted in bibliometric studies encompass co-authorship, co-citation, and co-occurrence analysis. Specifically, co-authorship analysis aims to investigate the relationships among authors, nations, or institutions, predicated on the quantity of jointly produced papers. Co-occurrence analysis employs a quantitative methodology to explore the relationship between different elements, based on their concurrent appearances. Co-citation analysis reveals the relational strength of cited elements by considering the number of elements that cite them.

The Online Analysis Platform of Literature Metrology was employed for conducting co-authorship and publication analyses across various countries/regions. VOSviewer serves as a software tool for the creation and visualization of bibliometric networks, which may be formulated leveraging citations, common citations, or co-authorship collaborations (18). In the map instantiated by VOSviewer, each node symbolizes an element. In the context of this study, VOSviewer facilitated the analysis of clustering patterns among countries, institutions, authors, and journals, taking into account the number of publications, citations, and the linkage potency of each constituent. A wide link width between nodes signifies a robust degree of cooperation, and larger nodes denote a higher number of reflections. Concurrently, CiteSpace served as an invaluable tool for bibliometric analysis (19, 20). Utilizing CiteSpace, we assessed keyword/reference clustering and timelines, as well as the centrality of countries, institutions, authors, and keywords. CiteSpace parameters were set as follows: (1) Time slice from 2003 to 2023, with each slice representing one year; (2) Single node type selection at a time; (3) Selection criteria were based on the g-index, with *k* = 15; (4) Pruning was performed via the pathfinder method. Moreover, Microsoft Excel 2019 was deployed to illustrate the global output and developmental trend of relevant papers. Impact factor (IF) and category quartile data were derived from the Journal Citation Reports (JCR) 2022 (21). The H-index is a composite index that serves as a significant metric for evaluating the quantity and quality of academic output produced by a scientific researcher, country, journal, or institution.

### Research ethics

The data sources utilized in our study were derived from publicly accessible databases. As such, obtaining approval from an ethics committee was deemed unnecessary.

## Results

### General information

#### Analysis of publications

**Figure 2:**
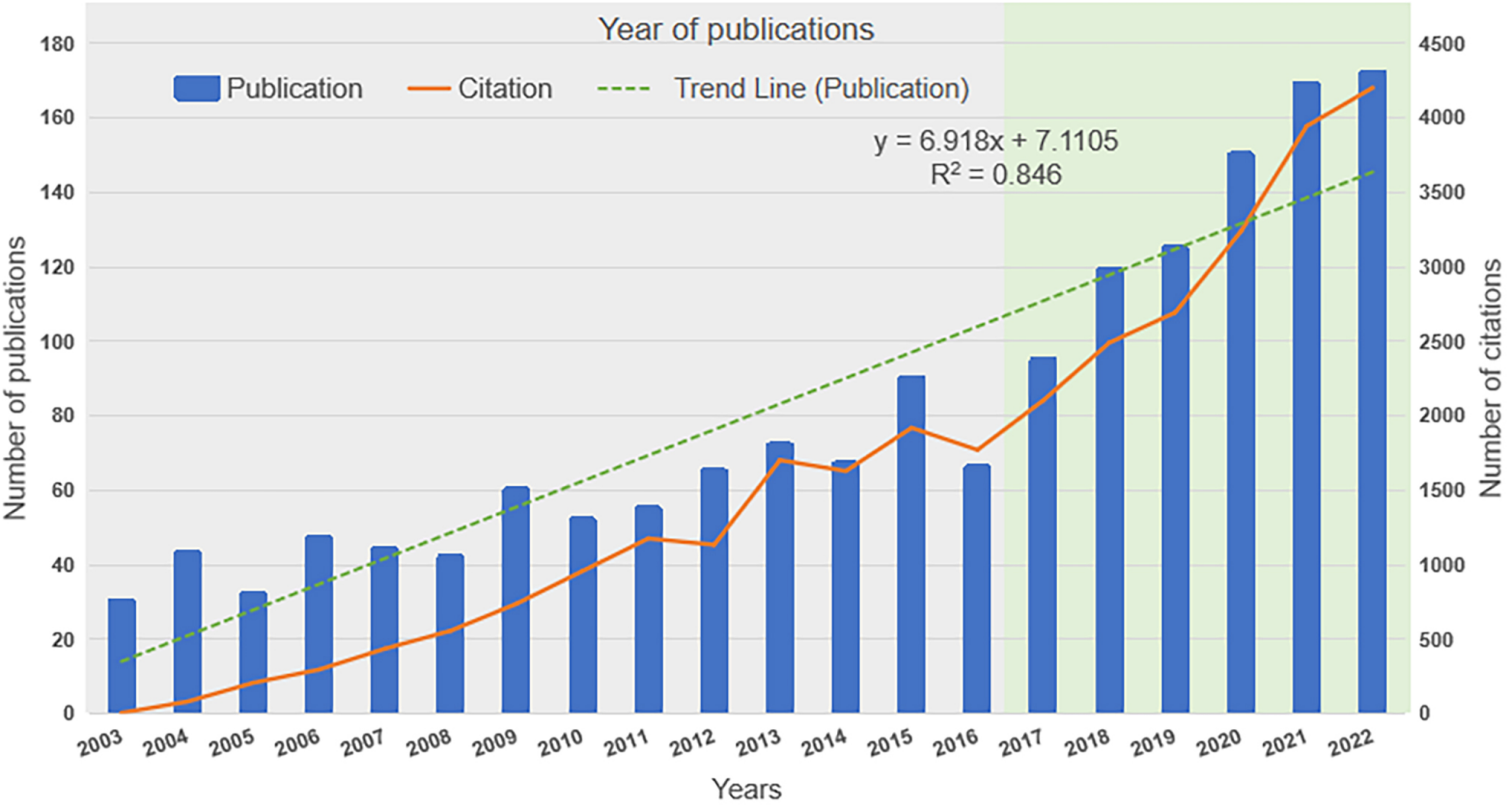
The number of publications and total citations.

A total of 1,643 publications focused on exercise for hypertension research were identified between Jan 1st, 2003 and May 18th, 2023, including 1,485 articles and 158 reviews. The average annual output was 83. As presented in Figure 2, the field of exercise for hypertension research exhibited a fluctuating publication output from 2003 to 2016, with an overall upward trend since 2017. The sustained increase in publications after 2017 likely reflects the growing recognition of exercise as a non-pharmacological intervention for hypertension prevention and management. The rising volume of publications on exercise for hypertension indicates that more studies are being conducted in this field, leading to the generation of new knowledge. The increasing interest in this topic might also indicate a recognition of the importance of non-pharmacological interventions for hypertension. Moreover, the 1,643 publications in this research field were cited a total of 32,491 times from 2003 to 2023. The number of citations increased from 5 in 2003 to 4,203 in 2022. The increase in citation rates over time indicates that the findings and insights produced by these studies are significant and relevant to the scientific community.

#### Analysis of countries/regions

**Figure 3:**
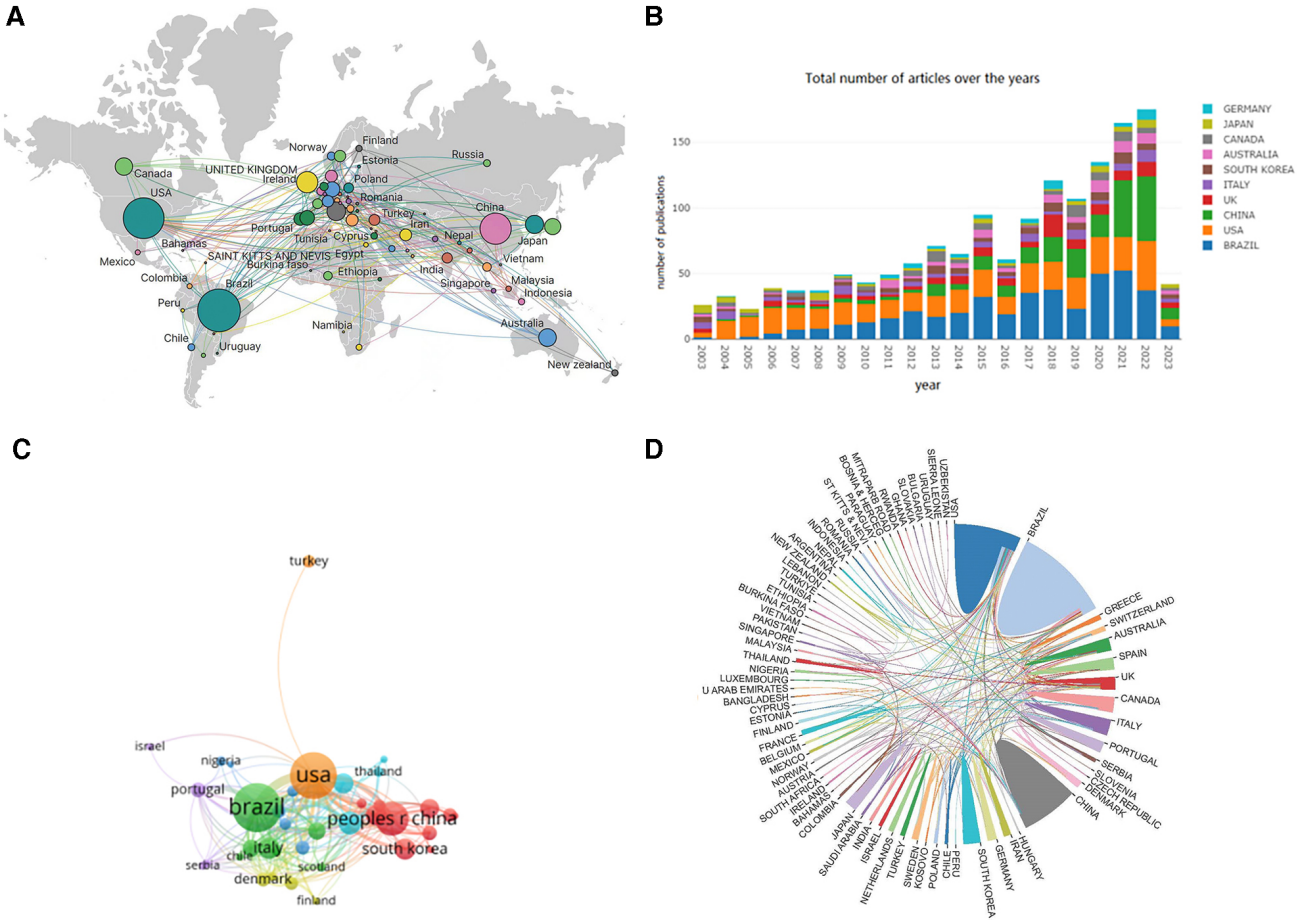
Visual map of countries/region. (**A**) Geographic distribution map based on the total publications of different countries/regions. (**B**) Number of annual publications and growth trends of the top 10 countries/regions. (**C**) Collaboration network analysis of the countries/regions. (**D**) The international network cooperation between the countries/regions.

Publications were published in 80 different countries/regions (Figure 3). Table 1 lists the top 10 most productive countries/regions, with Brazil (*n* = 420) leading, followed by the USA (*n* = 382), and China (*n* = 197). The USA had the highest citation count (9,414 times) and H-index = 49. Figure 3A shows a concentration of publications in North America, South America, Europe, and East Asia. Notably, China exhibited a substantial increase in publication output in 2021 (Figure 3B); this may be due to increased investment in research and development, an aging population, and high rates of hypertension prevalence (22–24). The cooperation network between countries and regions is depicted in Figures 3C,D, indicating that the USA collaborates with various countries, particularly Brazil and China. Total link strength (TLS) measures global co-authorship and is represented by the width of the lines connecting nodes. The visual co-authorship map reveals that the top three countries in terms of TLS are the USA, Brazil, and England. Beyond the USA, collaboration among other nations is limited. This underscores the need for fostering international partnerships to further advance exercise for hypertension research. Collaboration can pool resources, expertise, and knowledge from various regions to conduct more comprehensive and impactful studies.

**Table 1:** Top 10 countries in the field of exercise for hypertension.

Rank	Countries	Counts	Citations	TLS	H-index	Centrality
1	Brazil	420	6,359	143	38	0.23
2	USA	382	9,414	222	49	0.47
3	China	197	2,243	74	27	0.06
4	England	92	1,682	121	25	0.35
5	Italy	80	1,299	49	21	0.16
6	South Korea	77	1,112	29	19	0.02
7	Australia	72	1,515	60	22	0.07
8	Canada	70	1,967	40	25	0.13
9	Japan	60	954	22	9	0.07
10	Germany	52	1,288	47	19	0.06

#### Analysis of institutions

**Figure 4:**
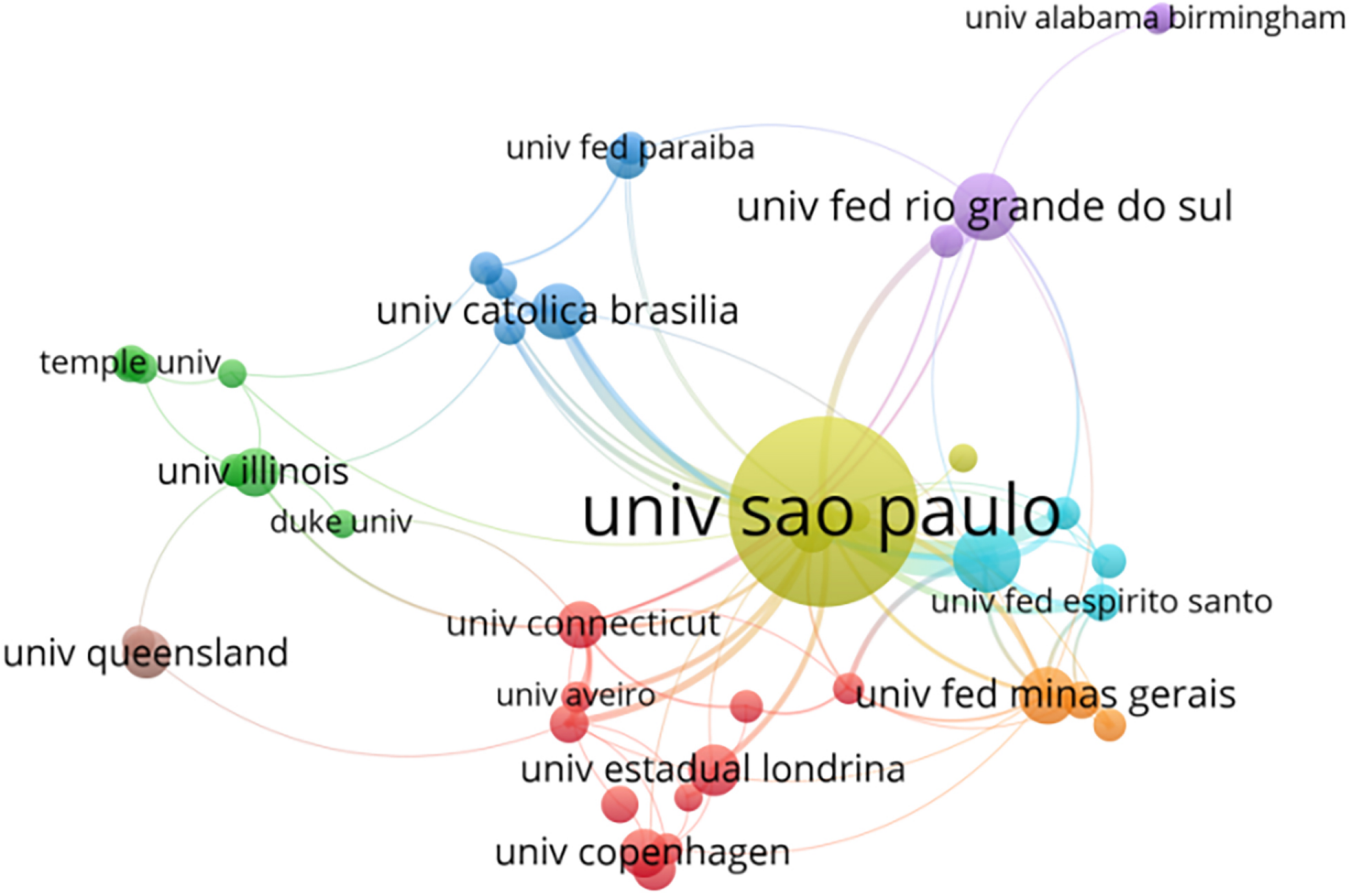
Collaboration network analysis of institutions.

Publications originated from 2197 different institutions (Figure 4). Table 2 lists the top 10 most productive institutions. The University of Sao Paulo published the most papers (*n* = 139), followed by Federal University of Sao Paulo (*n* = 32) and Universidade Federal do Rio Grande do Sul (*n* = 32).

**Table 2:** Top 10 institutions in the field of exercise for hypertension.

Rank	Institution	Country	Counts	Citations	TLS	H-index	Centrality
1	University of Sao Paulo	Brazil	139	3,208	132	34	0.08
2	Federal University of Sao Paulo	Brazil	32	526	46	15	0.17
3	Universidade Federal do Rio Grande do Sul	Brazil	32	662	42	16	0.00
4	The Federal University of Minas Gerais	Brazil	26	432	37	14	0.07
5	University Catholic of Brazil	Brazil	25	596	33	12	0.13
6	Universidade Estadual de Londrina	Brazil	22	258	20	9	0.06
7	University of Illinois	USA	21	877	32	15	0.02
8	University of Copenhagen	Denmark	21	586	19	15	0.06
9	University of Queensland	Australia	21	612	15	14	0.21
10	University of Connecticut	USA	20	689	37	12	0.08

#### Analysis of journals

Over the past 20 years, 280 academic journals published articles related to exercise for hypertension. The top 10 journals published 23.25% (*n* = 382) of these papers, as shown in Table 3. The average IF of the top 5 journals is 5.914. *Hypertension* (*n* = 48, IF = 9.897), a journal from the USA, had the highest IF among these journals. It focuses on basic science, clinical treatment, and prevention of hypertension and related cardiovascular, metabolic and renal diseases. The articles published in *Hypertension* are expected to remain significant for years to come. In terms of publications volume, the top three journals were *Journal of hypertension* (IF = 4.776), *Hypertension* (IF = 9.897), and *Hypertension research* (IF = 5.528). The *Journal of Hypertension*, based in the USA, focuses on research related to the basic science, clinical treatment, and prevention of hypertension, as well as associated cardiovascular, metabolic, and renal diseases. Given the quality and scope of its published papers, they are expected to remain significant for years to come.

**Table 3:** Top 10 journals in the field of exercise for hypertension.

Rank	Journals	Counts	Citations	H-index	IF (2022)	JCR quartile
1	Journal of hypertension	59	832	17	4.776	Q1
2	Hypertension	48	1,573	20	9.897	Q1
3	Hypertension research	47	689	12	5.528	Q1
4	Clinical and experimental hypertension	44	296	10	2.088	Q3
5	American journal of hypertension	40	773	14	3.080	Q2
6	Journal of human hypertension	35	441	14	2.877	Q2
7	Frontiers in physiology	32	156	7	4.755	Q2
8	Journal of clinical hypertension	28	329	10	2.885	Q2
9	International journal of environmental research and public health	26	157	6	4.614	Q1
10	Journal of applied physiology	23	290	9	3.880	Q1

#### Analysis of authors

**Figure 5:**
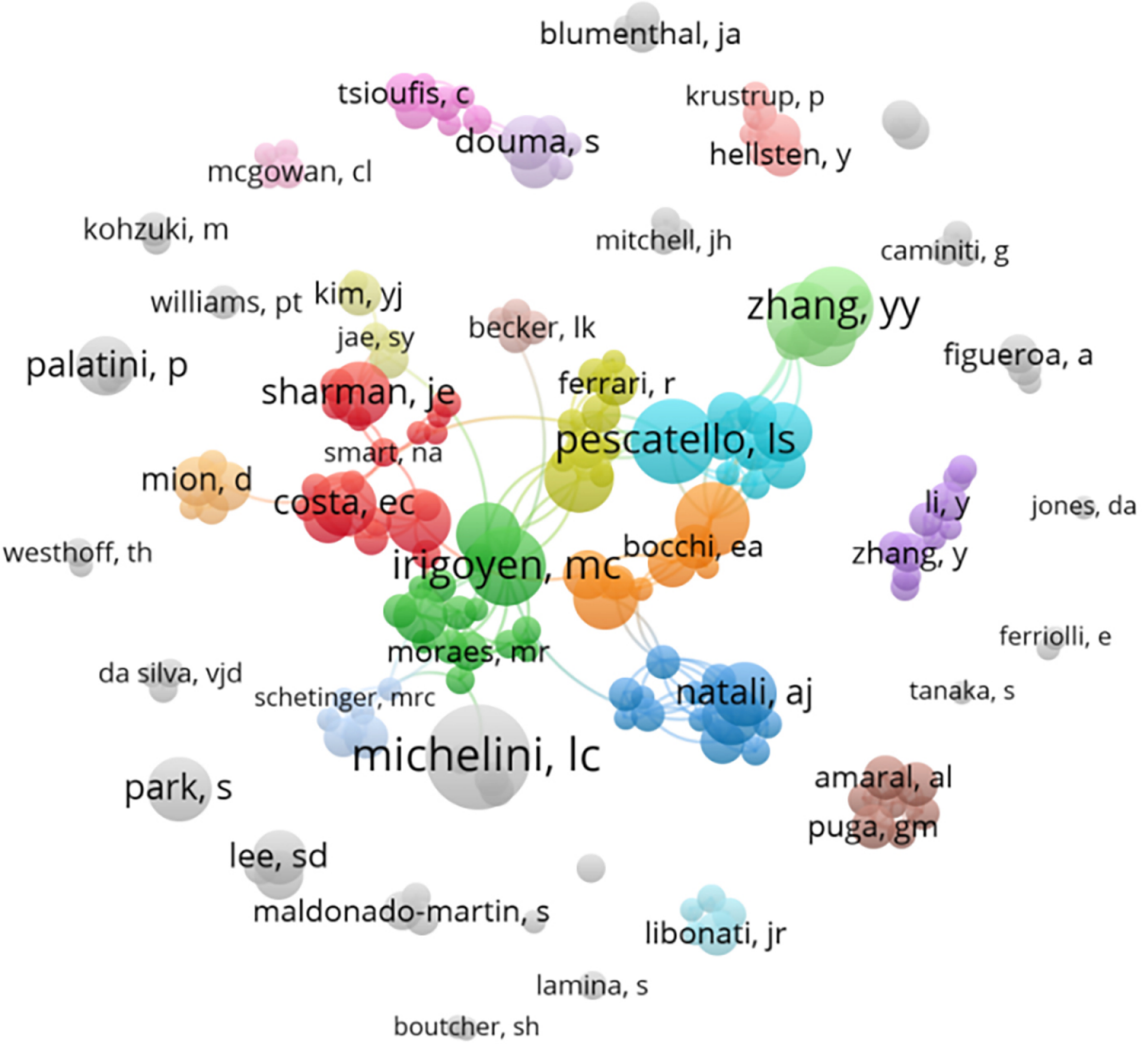
Collaboration network analysis of authors.

As many as 8,145 authors contributed to the research on exercise for hypertension. Table 4 presents the top 10 authors in exercise for hypertension research, with Maria Claudia C. Irigoyen ranking first (*n* = 18), followed by Linda S. Pescatello (*n* = 17), Li-jun Shi (*n* = 15). The cooperation map (Figure 5) indicates that the scale of collaboration among authors is relatively light, suggesting a need for increased overall connections between researchers in this field.

**Table 4:** Top 10 authors in the field of exercise for hypertension.

Rank	Author	Counts	Citations	TLS	H-index	Country	Institution
1	Maria Claudia C. Irigoyen	18	340	25	11	Brazil	Universidade de Sao Paulo
2	Linda S. Pescatello	17	1,713	51	11	USA	University of Connecticut
3	Li-jun Shi	15	154	32	9	China	Fudan University
4	Claudia L. M. Forjaz	15	324	20	9	Brazil	University of São Paulo
5	Alexandre M. Lehnen	14	131	27	6	Brazil	Fundação Universidade de Cardiologia
6	Guilherme V. Guimarães	14	622	55	11	Brazil	Instituto do Coração do Hospital
7	James Sharman	13	374	19	11	Brazil	University of Tasmania
8	Antonio José Natali Marcos	13	149	49	7	Brazil	Universidade Federal de Viçosa
9	Polito Thales	13	133	40	7	Brazil	Universidade Estadual de Londrina
10	Nicolau Primo NP Gomes	12	142	48	7	Brazil	Universidade Federal de Viçosa

### Research hotspots

#### Analysis of co-cited journals

The top 10 most-cited journals are displayed in Table 5. A journal's influence in its respective field is determined by the frequency of co-citations, serving as a measure of its significance. Notably, nine journals in Table 5 have been cited over 1,000 times. The three most frequently co-cited publications were *Hypertension* (IF = 9.897), *Circulation* (IF = 39.918), and *Annals of Internal Medicine* (IF = 51.598).

**Table 5:** Top 10 Co-cited journals in the field of exercise for hypertension.

Rank	Journals	Country	Citations	TLS	JCR quartile	IF (2022)
1	Hypertension	USA	4,206	1,75,831	Q1	9.897
2	Circulation	USA	2,859	1,34,912	Q1	39.918
3	Annals of Internal Medicine	USA	2,309	1,07,842	Q1	51.598
4	Medicine and Science in Sports and Exercise	USA	2,010	86,709	Q1	6.29
5	Journal of Applied Physiology	USA	1,536	69,997	Q2	3.880
6	American Journal of Hypertension	USA	1,253	64,058	Q2	3.080
7	Journal of the American College of Cardiology	USA	1,090	48,485	Q2	6.106
8	Lancet	USA	1,046	44,257	Q1	202.731
9	American Journal of Physiology-heart and Circulatory Physiology	USA	1,027	46,038	Q1	5.125
10	Journal of the American Medical Association	USA	973	47,731	Q1	151.335

#### Analysis of co-cited authors

**Figure 6:**
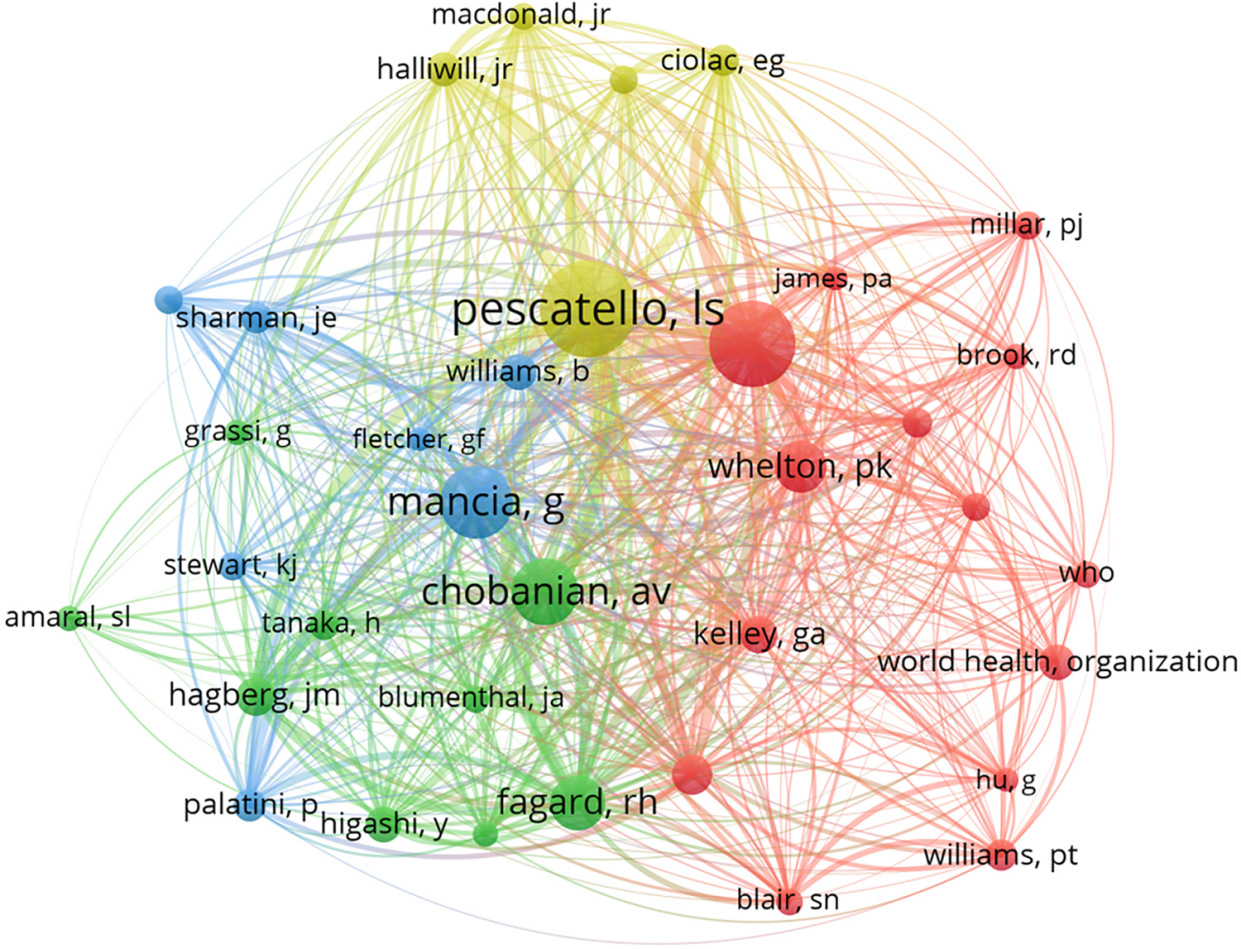
The network visualization map of co-cited authors.

The network map of co-cited authors is shown in Figure 6. Linda S. Pescatello ranked first with 482 citations, followed by Cornelissen Véronique A, Mancia Giuseppe, and Chobanian Aram V with 411, 325, and 297 citations, respectively (Table 6). The top 10 co-cited authors collectively exceeded 2,000 citations, highlighting their prominence and influence in the domain of exercise for hypertension research.

**Table 6:** Top 10 Co-cited authors in the field of exercise for hypertension.

Rank	Author	Citations	TLS	Country	Institution
1	Linda S. Pescatello	482	4,602	USA	University of Connecticut
2	Cornelissen, Véronique A	411	3,887	Belgium	Catholic University of Leuven
3	Mancia, Giuseppe	325	2,679	Italy	Centro di Fisiologia Clinica e Ipertensione
4	Chobanian, Aram V	297	2,165	USA	Boston University School of Medicine
5	Fagard, R H	222	2,533	Belgium	University of Leuven
6	Whelton, Paul K	204	1,721	USA	University of Tulane
7	Whelton, Seamus P	142	1,471	USA	University of Tulane
8	Hagberg, J M	138	1,646	USA	University of Maryland
9	Kelley, G A	130	1,854	USA	University of Northern Illinois
10	Williams, Bryan	126	1,009	UK	University of Leicester

#### Analysis of co-cited references

**Figure 7:**
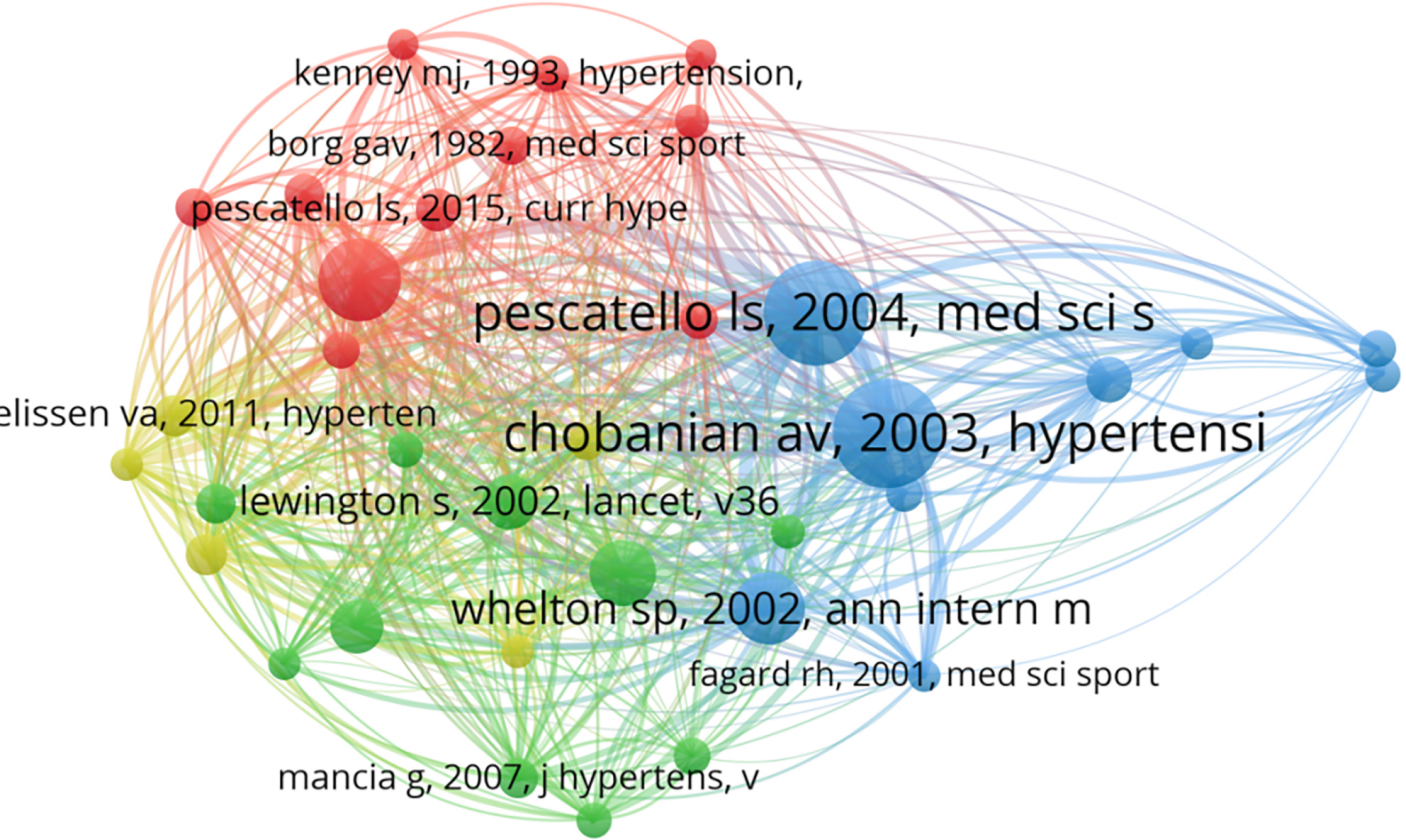
The network visualization map of co-cited references.

Reference co-citation analysis determines highly co-cited references that are frequently cited together by other articles, making it a common approach for exploring research foci in a given academic field. The network map of co-cited references is displayed in Figure 7, and Table 7 lists the top ten articles according to number of citations. We concluded that these ten articles concentrate on 3 research themes: (1) exercise interventions for hypertension prevention and treatment, (2) age-specific relevance of exercise for hypertension, (3) the global burden of hypertension and the role of exercise.

**Table 7:** Top 10 references in the field of exercise for hypertension research from 2003 to 2023.

Rank	Co-cited reference	Journal	Years	Co-cited counts	JCR quartile	IF (2022)
1	The seventh report of the Joint National Committee on prevention, detection and treatment of high blood pressure	Hypertension	2003	252	Q1	9.897
2	Exercise and hypertension	Medicine & Science In Sports & Exercise	2004	240	Q1	6.29
3	Exercise training for blood pressure: a systematic review and meta-analysis	Journal of The American Heart Association	2013	169	Q2	6.106
4	Effect of aerobic exercise on blood pressure: a meta-analysis of randomized, controlled trials	Annals of Internal Medicine	2002	139	Q1	51.598
5	Effects of endurance training on blood pressure, blood pressure-regulating mechanisms, and cardiovascular risk factors	Hypertension	2005	120	Q1	9.897
6	Age-specific relevance of usual blood pressure to vascular mortality: a meta-analysis of individual data for one million adults in 61 prospective studies	Lancet	2002	91	Q1	202.731
7	Global burden of hypertension: analysis of worldwide data	Lancet	2005	88	Q1	202.731
8	The role of exercise training in the treatment of hypertension: an update	Sports Medicine	2000	70	Q1	11.928
9	Exercise for Hypertension: A Prescription Update Integrating Existing Recommendations with Emerging Research	Current Hypertension Reports	2015	65	Q3	3.036
10	Impact of resistance training on blood pressure and other cardiovascular risk factors: a meta-analysis of randomized, controlled trials	Hypertension	2011	61	Q1	9.897

#### Analysis of keywords co-occurrence

**Figure 8:**
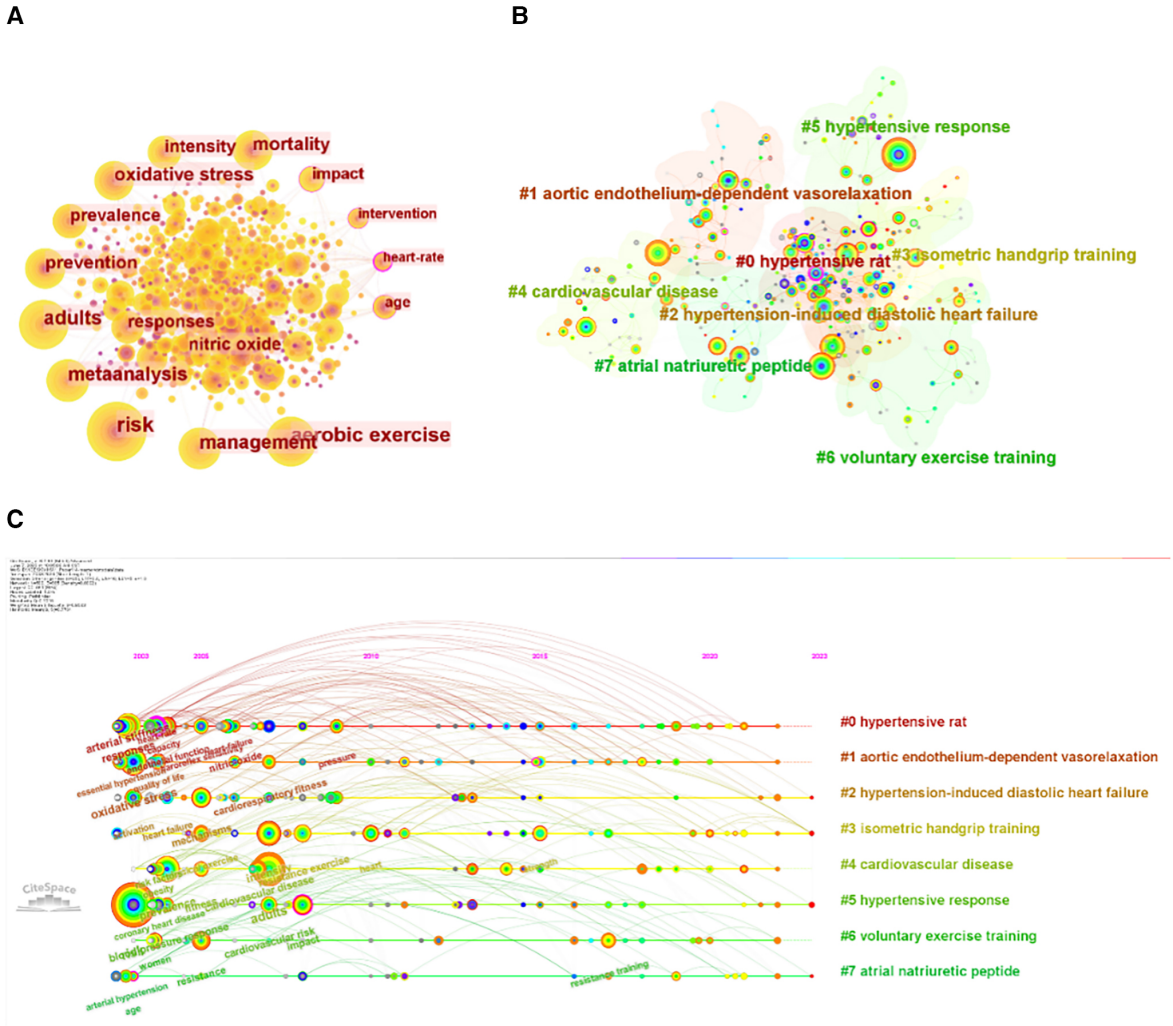
Analysis of keywords. (**A**) Keywords co-occurrence. (**B**) Keyword clustering. (**C**) Timeline of keywords.

Keywords summarize the core material of a paper in great detail. Table 8 reports the top 20 keywords, while Figures 8A,B illustrate the co-occurrence and cluster analysis of these keywords, respectively. Within the exercise for hypertension research, significant research areas encompass epidemiological information (e.g., risk, mortality, prevalence), types of exercise (e.g., aerobic exercise, resistance exercise), target population (e.g., adults, men), mechanism (e.g., adrenergic blockade, oxidative stress), and study design (e.g., meta-analysis). To display temporal trends, a timeline plot was constructed (Figure 8C). In the field of exercise for hypertension, a total of seven clusters have been identified, with “#2 hypertension-induced diastolic heart failure,” “#3 isometric handgrip training,” “#5 hypertensive response,” and “#7 atrial natriuretic peptide” emerging as recent research hotspots.

**Table 8:** Top 20 keywords in the field of exercise for hypertension.

Rank	Keywords	Occurrences	Centrality	Rank	Keywords	Occurrences	Centrality
1	Risk	244	0.01	11	Intensity	81	0.09
2	Aerobic exercise	180	0.01	12	Response	80	0.03
3	Adults	167	0.02	13	Prevalence	80	0.03
4	Adrenergic blockade	131	0.01	14	Men	75	0.05
5	Meta-analysis	131	0.01	15	Arterial stiffness	74	0.05
6	Management	126	0.03	16	Postexercise hypotension	69	0.03
7	Oxidative stress	115	0.06	17	Mechanisms	69	0.02
8	Mortality	109	0.04	18	Cardiovascular disease	62	0.01
9	Prevention	106	0.07	19	Resistance exercise	60	0.05
10	Nitric oxide	82	0.05	20	Guideline	57	0.10

### Global trends of exercise for hypertension research

#### Analysis of keywords with the strongest citation bursts

**Figure 9:**
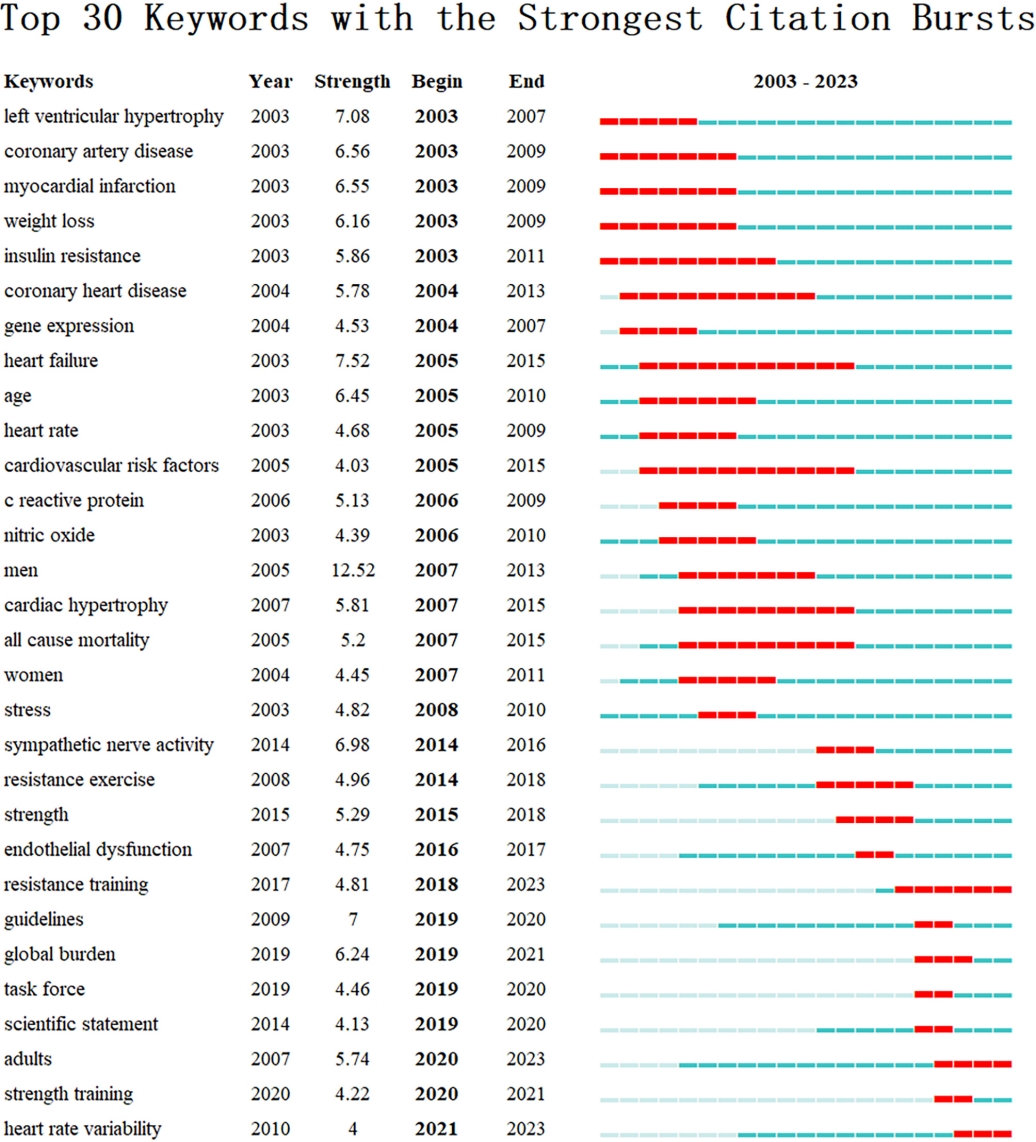
Visualization map of top 30 keywords with the strongest citation bursts.

Since 2003, a total of 30 keywords have experienced significant bursts. The results of burst detection are illustrated in Figure 9, showing that keywords such as “resistance training”, “adults”, “heart rate variability” gained prominence in recent years. This indicates that research concerning these topics is currently garnering attention and signifies potential developmental trends in the field of exercise for hypertension research.

#### Analysis of co-cited references

**Figure 10:**
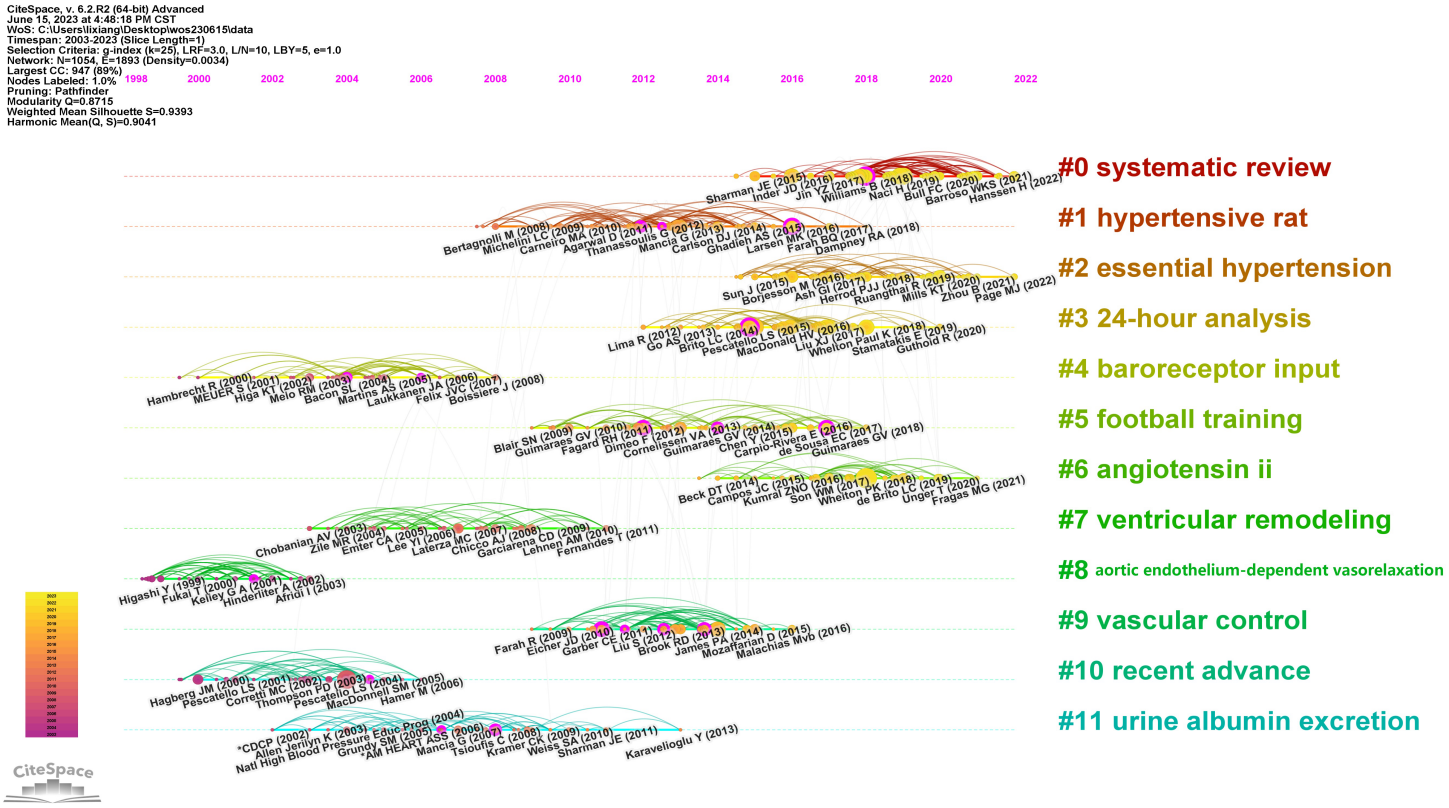
Timeline of co-cited reference.

The timeline view of co-cited references serves as a visual diagram reflecting the temporal characteristics of research hotspots in this field. Among the 12 clusters depicted in Figure 10, the #8 cluster, focusing on aortic endothelium-dependent vasorelaxation, emerged as the earliest research hotspot. Currently, the most prevalent research hotspots are #0 systematic review and #2 essential hypertension.

## Discussion

### General information

The research on exercise for hypertension has been extensively studied over the last two decades. The subject has been researched in 80 countries, with eight developed and only two developing countries among the top 10 countries studied. Despite ranking second in published papers, the USA has the highest centrality score, indicating its leadership in this research area. This can be attributed to the country's high prevalence of hypertension and substantial investment in exercise treatment for hypertension. Brazil ranks first in the number of articles published on this topic. This may be associated with the high incidence of hypertension in the country and the low cost of exercise treatment (25). China follows closely in third place, which may also be credited to the high incidence of hypertension and the popularity of traditional Chinese practices such as Baduan Jin and Tai Chi (26, 27). The top 10 institutions make up 21.85% of total publication yields, indicating significant academic accomplishments. Brazil's University of Sao Paulo, the country's leading comprehensive university, is the top-ranked institution, closely working with the fifth-ranked University Catholic of Brazil.

A total of 280 diverse academic journals published articles related to exercise for hypertension research, with the top 10 journals accounting for 23.25% of the publications. Among these, the *Journal of Hypertension* was the most frequently published journal, while it was challenging to publish relevant papers in high impact factor journals. The identification of crucial authors can aid investigators in finding potential collaborators. Irigoyen Maria Claudia C, Linda S. Pescatello, and Shi Li-jun were the most productive and influential authors in this area. Irigoyen Maria Claudia C is a researcher from the Universidade de Sao Paulo whose work focuses on exploring the relationship between physical activity, heart health, and hypertension. Her research supports the use of exercise therapy as a complementary approach to managing hypertension and improving overall cardiovascular health (28, 29).

### Research hotspots

According to the co-cited authors, Linda S. Pescatello from USA is the top-ranked author for co-citations. In her most recent work, she discovered that, in individuals with resistant hypertension, a 12-week aerobic exercise regimen resulted in 24-hour and ambulatory daytime blood pressure, as well as office systolic blood pressure. Her discovery strengthens the evidence base advocating for the integration of moderate-intensity aerobic exercise as a standard adjunctive therapy for this patient demographic (30, 31). The highest co-cited reference was published by Linda S. Pescatello in 2003 in the journal *Hypertension* (32). This paper reported that a healthy lifestyle can lower blood pressure, with exercise being an important way to promote recovery from hypertension.

Our analysis of co-occurrence keywords revealed five important research areas: epidemiological information, types of exercise, target population, mechanism, and study design. Hypertension epidemiology has been a hot topic due to its increasing global incidence; the latest data reveals that hypertension affects 34% of the world's population (32, 33). It is a significant risk factor for cardiovascular disease(CVD), chronic kidney disease, dementia, and other major illnesses. Aerobic and resistance exercises are currently the most popular methods used to intervene in hypertension. Exercise training results in considerable reductions in all hypertension blood pressure measures in individuals with hypertension (34). While further evidence is necessary to determine whether exercise can replace anti-hypertensive drugs, exercise training, particularly using aerobic modalities, appears to be an effective adjunctive therapy treatment for hypertension (35). Resistance exercise training has also been shown to reduce blood pressure (9, 36). Among the target population, hypertensive patients are prevalent among adults with men older than 50 years at higher risk for hypertension (37, 38).

The mechanism of exercise in treating hypertension is a research hotspot, particularly regarding adrenergic blockade and oxidative stress. Adrenergic blockade is one of the most important drug treatments for hypertension and heart disease. It can inhibit the effects of adrenaline on the body via β-blockers or *α*-blockers, which slow the heart rate, dilate blood vessels, and lower blood pressure (39, 40). Combined with exercise, adrenergic blocking drugs can synergistically control hypertension. During aerobic exercise, adrenaline secretion is suppressed, achieving better blood pressure-lowering effects. Additionally, aerobic exercise reduces the level of adrenaline in the blood (41). Furthermore, numerous studies have shown that exercise can reduce oxidative stress and inflammatory responses, leading to blood pressure reduction, and improvements in vascular function (42, 43). The hormone Atrial Natriuretic Peptide (ANP), currently a pivotal area of research, is secreted by the heart, playing a significant role in the regulation of processes such as blood pressure and fluid balance. Studies indicate that ANP levels often decrease in patients with hypertension. This reduction could stem from cardiovascular alterations caused by hypertension, hampering ANP secretion or perhaps due to adaptive bodily mechanisms causing a decrease in ANP levels. Conversely, exercise has demonstrated potential in enhancing ANP levels by stimulating ANP production via increased cardiac output and heightened Sympathetic Nervous System activity. This amelioration leads to a reduction in blood pressure and assists in balancing body fluid (44–46).

Meta-analysis is a systematic and statistical method for integrating existing studies that combines the results of independent studies to evaluate the overall effect of a specific variable or intervention. It is commonly used in exercise for hypertension research due to the large number of studies, variability in findings, ability to assess effect sizes, and development of appropriate guidelines and recommendations. By combining multiple studies, it improves the reliability and accuracy of overall studies, allowing physicians and the public to better understand how to prevent and treat hypertension. Since 2020, 55 meta-analyses on exercise interventions for hypertension have been published. One of the highest IF meta-analyses was published in the *British Journal of Sports Medicine* (IF = 18.479) (47). This study revealed that short-duration exercise of any type can reduce the risk of all-cause mortality and serious adverse events in patients with hypertension, type 2 diabetes, or CVD.

### Global trends

Research frontier themes are frequently indicated by keywords with citation bursts. A thorough examination of the most recent keyword bursts revealed three new trends in exercise for hypertension research. These are what they are:

I. Resistance training: Resistance training, also referred to as strength or weight training, is a type of exercise that primarily aims to develop and strengthen skeletal muscles. Its positive effect on hypertension has been well-documented in several studies, which have shown a decrease in blood pressure among individuals with hypertension who engage in resistance training. This can be attributed to the improvement of arterial compliance, vascular function, and reduction of arterial stiffness, all of which contribute to better blood flow and lower blood pressure (48, 49). Moreover, resistance training promotes muscle growth and overall cardiovascular health, further supporting its effectiveness as an adjunct therapy for hypertension (36). Combining resistance training with aerobic exercise may provide additional benefits for individuals with hypertension. Nonetheless, more research is required to determine the optimal frequency, intensity, and duration of resistance training for different populations with hypertension (9, 50, 51).
II. Adults: The World Health Organization defines individuals aged 18 years and older as adults, a group that is differentiated from children and adolescents in the fields of medicine and public health due to varying health concerns and risks. Adults are more susceptible to chronic diseases, including hypertension, which poses a significant economic and public health burden. Improving lifestyles, particularly through exercise, among young people is crucial in preventing age-related increases in blood pressure. Management strategies for hypertension in older adults must consider factors such as frailty, complex medical comorbidities, and psycho-social factors on an individual basis. Non-pharmacological lifestyle interventions should be encouraged to reduce the risk of developing hypertension and to serve as adjunctive therapy to lower the need for medications (36, 37, 49).
III. Heart rate variability (HRV): HRV refers to the variation in the time interval between consecutive heartbeats, also known as the change in the speed of the heartbeat. Higher HRV is generally associated with better cardiovascular function and resilience, while lower HRV is linked to a higher risk of anxiety and depression, as well as increased mortality from CVD (52). Regular exercise, particularly endurance training, has been found to improve HRV levels. In a study involving middle-aged hypertensive women, combined aerobic and resistance training resulted in significant improvements in HRV parameters (53). As such, exercise programs that incorporate both aerobic and resistance training may be beneficial for the management of hypertension by enhancing cardiac autonomic control (54).

## Limitation

There are several limitations to note in this study. Firstly, we only utilized WOSCC as our database, which may have led to the omission of relevant papers from other databases. Secondly, there is a possibility that significant non-English papers were overlooked, resulting in research bias and a reduction in credibility. Finally, due to the constant updates of the database, recently published high-quality articles may have been underestimated because of their inadequate citations.

## Conclusion

The analysis of literature on exercise for hypertension from 2003 to 2023 shows an increasing interest, with major contributions from the US and University of São Paulo. Publications primarily come from Brazil, USA, and China, underscoring the importance of international collaboration in this domain. Exercise is gaining prominence as an essential non-pharmacological means to prevent and manage hypertension. Notable authors include Maria Claudia C. Irigoyen and Linda S. Pescatello. Increasing author collaborations are observed, though further cooperation is needed. Key themes include hypertension prevention and treatment through exercise, age-specific relevance, and global impact. New trends focus on “hypertension-induced diastolic heart failure” and “isometric handgrip training.” This bibliometric review offers valuable insights on emerging trends and future research direction in exercise for hypertension.
