# Supplementary figures and images for: Trends in exercise for hypertension: a bibliometric analysis
Source: Front Cardiovasc Med. 2023 Oct 23;10:1260569. doi: 10.3389/fcvm.2023.1260569 (PMC10627159; doi:10.3389/fcvm.2023.1260569)

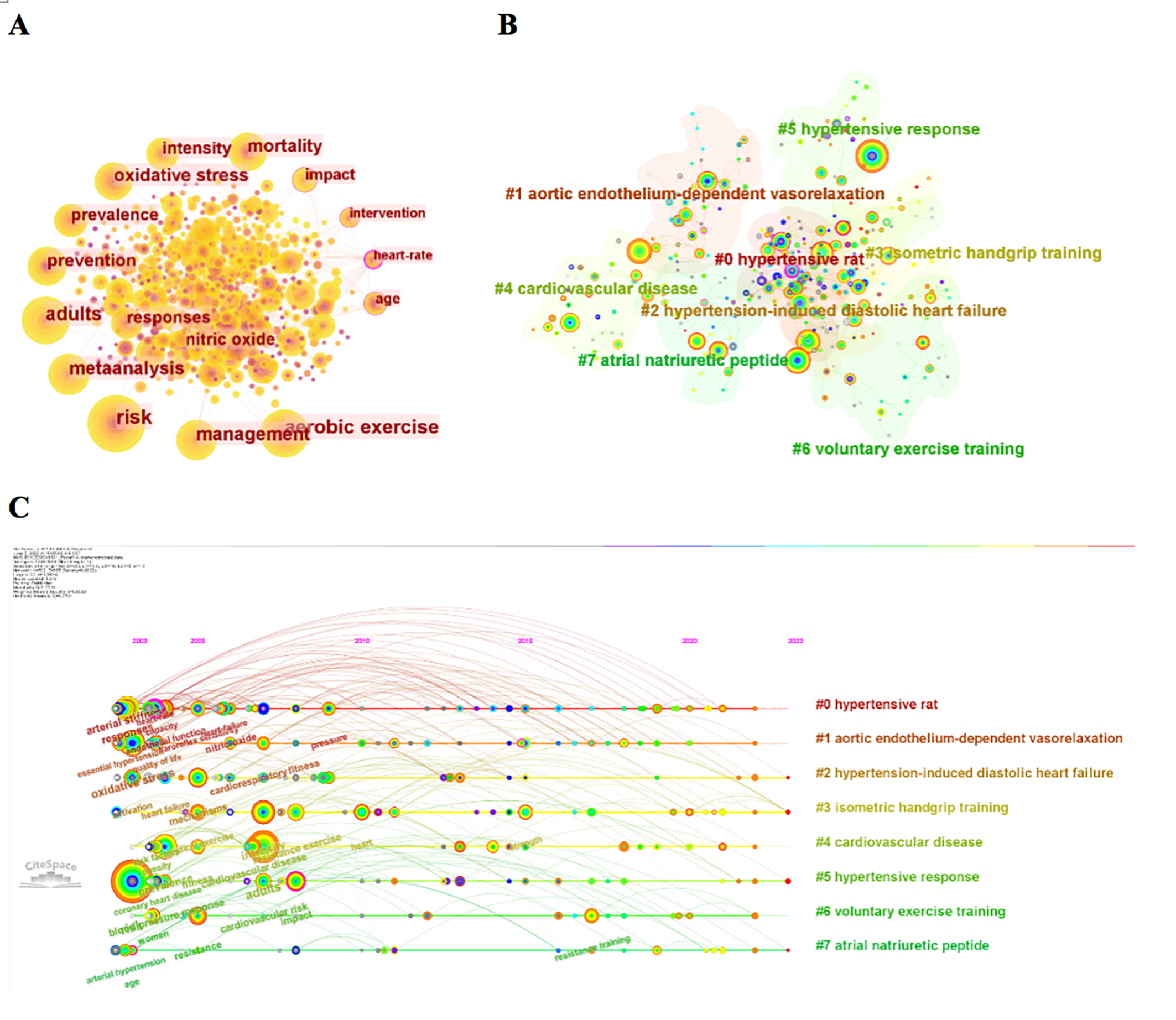

Supplement: Supplementary file 1 [file Image1.jpeg]

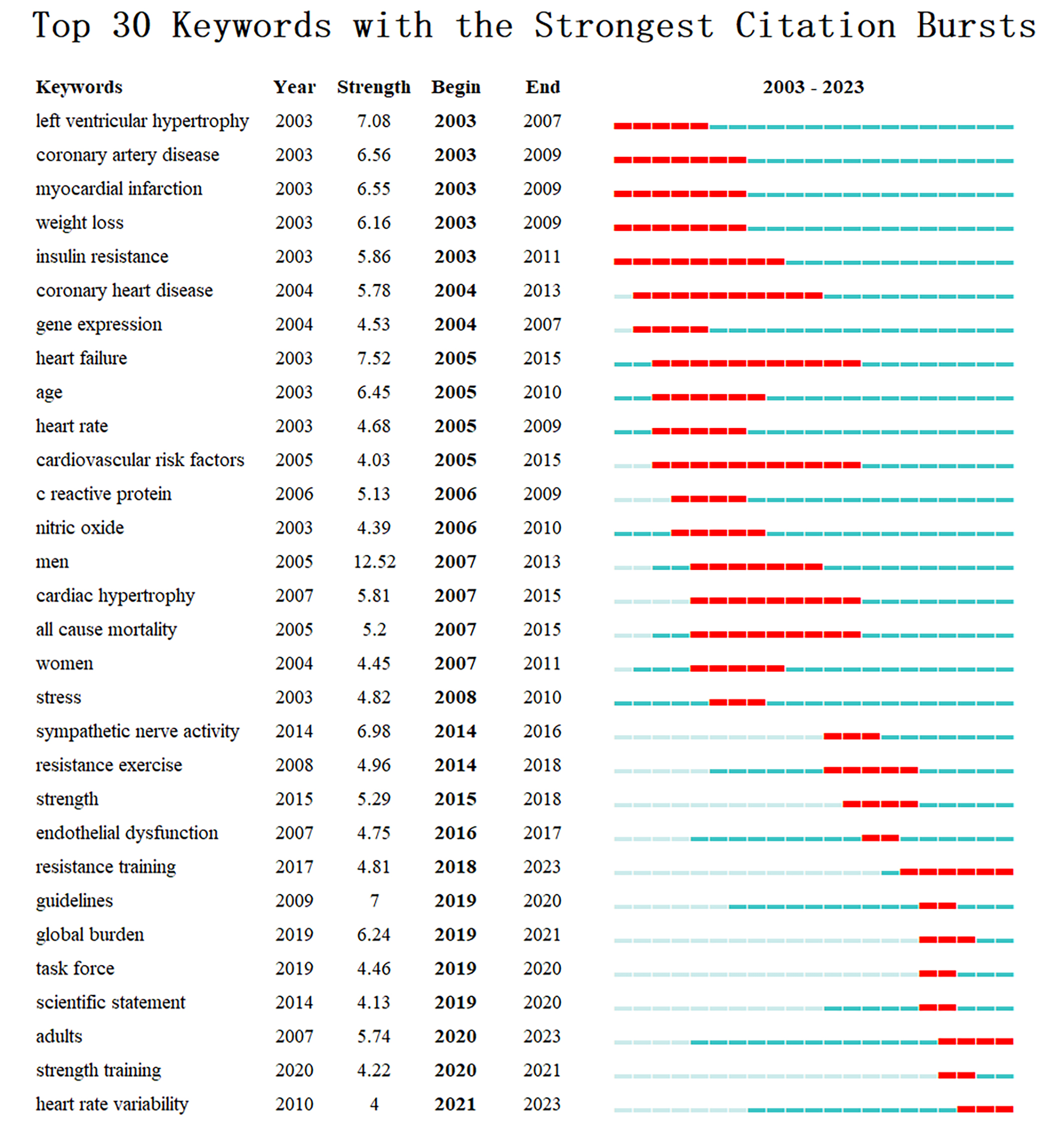

Supplement: Supplementary file 2 [file Image2.jpeg]

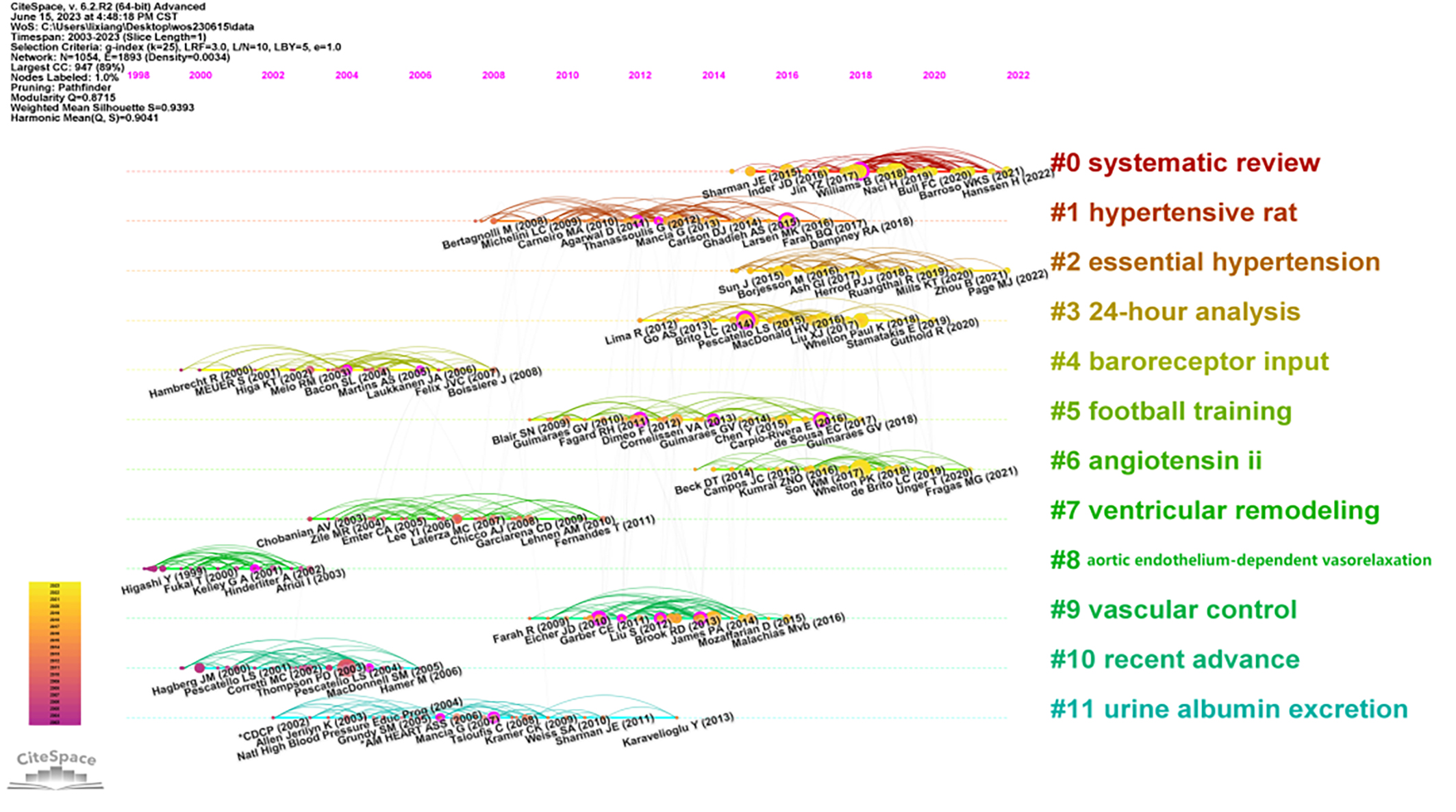

Supplement: Supplementary file 3 [file Image3.jpeg]
